# Editorial: Hypertension During Pregnancy and Future Risk of Cardiovascular and Other Long-Term Health Outcomes

**DOI:** 10.3389/fcvm.2020.569735

**Published:** 2020-10-15

**Authors:** Amanda Henry, Dexter Canoy

**Affiliations:** ^1^School of Women's and Children's Health, University of New South Wales, Kensington, NSW, Australia; ^2^Department of Womens and Childrens Health, St George Hospital, Sydney, NSW, Australia; ^3^George Institute for Global Health, University of New South Wales, Newtown, NSW, Australia; ^4^Deep Medicine, Oxford Martin School, University of Oxford, Oxford, United Kingdom; ^5^Nuffield Department of Women's and Reproductive Health, University of Oxford, Oxford, United Kingdom; ^6^NIHR Oxford Biomedical Research Centre, Oxford University Hospitals NHS Foundation Trust, Oxford, United Kingdom

**Keywords:** preeclampsia, hypertensive disorders of pregnancy (HDP), cardiovascular disease, mental health, endothelial dysfunction, randomized controlled clinical trial (RCT), lifestyle behavior change, guidelines

“Hypertension during pregnancy and future risk of cardiovascular and other health outcomes” attracted a wide range of submissions across the spectrum of this topic, from consideration of underlying vascular mechanisms through to mental health, reviews of guidelines and existing intervention studies, protocols for trials in progress, and consideration of the importance of this area to low and middle income countries.

The catalyst for this special issue was the fact that mothers with hypertensive disorders of pregnancy (HDP), such as preeclampsia and gestational hypertension, have increased cardiometabolic health risks long after delivery. Melchiorre et al. remind us of the strength of the epidemiological evidence in this area, with both retrospective and prospective cohorts consistently finding 1.5–3 times increase in risk after HDP of cardiovascular conditions including essential hypertension, coronary artery disease, cerebrovascular disease, peripheral vascular disease, and heart failure. Adjustment for pre-existing risk factors attenuates but does not eliminate these relationships, which are strengthened in those who suffer early-onset or recurrent HDP.

Although the underlying mechanisms for CVD after HDP remain uncertain, Kirollos et al. provide some insights into pathophysiological links through their systematic review of vascular structure and function in preeclampsia and postpartum after preeclampsia. Collectively, the 59 included studies suggest impaired endothelial function during and after preeclamptic pregnancies compared to normotensive pregnancies, and structural abnormalities including increased carotid intima-media thickness and accelerated coronary calcification and plaque deposition. However, findings were not universal, and there was paucity of research beyond the first few years postpartum, underscoring the need for more studies in this area.

In addition to physical sequelae of HDP, mental health is a key consideration. Roberts et al. reviewed the literature on depression, anxiety, and post-traumatic stress disorder after HDP. Their 17 included publications did not consistently find associations between HDP and mental health disorders. However, depression, anxiety, and post-traumatic symptoms trended toward increased prevalence and severity after HDP, especially in women who had given birth preterm or had preeclampsia with clinically severe features. The authors note that routine screening for mental health disorders on all women in the postpartum period may be beneficial, and with additional emphasis after HDP to alert clinicians to the need for additional follow-up and referral.

Although most literature on health after HDP is based on high-income country populations, both the acute burden of HDP and its associated maternal and neonatal mortality and morbidity, and the burden of cardiometabolic disorders, fall disproportionately on low and middle-income countries (LMICs). Nagraj et al. review the long-term health implications of cardiometabolic pregnancy complications, both HDP and gestational diabetes, in LMIC, and consider research priorities. A multi-dimensional strategy is proposed, including well-designed experimental studies of novel technologies including *mHealth* to first identify pregnancy complications and then aid follow-up postpartum to reduce long-term cardiometabolic disease burden. They also remind us of the importance of complex social factors that impact women's health across the life-course, and the importance of improving equity and access in LMIC to health interventions, including essential medications.

As it is impossible for healthcare practitioners to read all the original research on their areas of practice, guidelines and protocols are essential to summarize the evidence and provide authoritative guidance. Gamble et al. examine what current national and international guidelines have to say about HDP and subsequent cardiovascular disease. They found 16 guidelines from 2010 onwards, mostly from either obstetric or cardiology organizations/societies, that mentioned follow-up after HDP. Of these, only half provided any recommendation beyond the immediate postpartum period, recommendations varied with few details regarding appropriate frequency and timing of monitoring, and all were based on very low to moderate evidence grade. As noted by the authors, a number of research questions related to appropriate timing and nature of follow-up and interventions after HDP to prevent CVD need to be answered to improve the evidence base on which guidelines are built.

Lack of guidance on health after HDP is also noted by Roth et al., from the perspective of what the knowledge gaps of both women and healthcare providers are in this space. Their scoping review identified 12 studies, including 402 women and 1,215 healthcare providers. Most studies found that both women and healthcare providers had limited or no knowledge about links between HDP and CVD, and that when women had knowledge, this was primarily through their own sourcing of information and not from their healthcare provider. For healthcare providers, primary enablers of knowledge were the availability and awareness of guidelines, underscoring the importance of improving guidelines regarding health after HDP.

What, then, is being done to improve women's health after HDP? Lui et al., Aldridge et al., and Taylor et al. all examine various aspects of this question. Lui et al. performed a systematic review of randomized controlled trials (RCT) less than 10 years postpartum after HDP, looking for trialed interventions to reduce cardiovascular risk. Only two reported RCTs were identified, with a total of 352 women, highlighting the limited evidence for any effective intervention. There is an indication that lifestyle interventions may be effective, but further evidence is required. Four RCTs that are in progress were also identified, which will add to the evidence base once reported, including Be Healthe for Your Heart, whose protocol is published by Taylor et al. in this special issue. Their pilot RCT is evaluating, in Australian women less than 4 years post-preeclampsia, the acceptability of an online web-based lifestyle behavior change intervention aimed at supporting changes in modifiable CVD risk factors such as poor diet, physical inactivity, and excess body weight. Also in an Australian population, in a socioeconomically disadvantaged setting, Aldridge et al. are recruiting, albeit not in a randomized fashion, women after severe pregnancy complications including HDP. Women are followed up face-to-face 6 months postpartum (with planned 18-month and 5-year further follow-up), using an education and counseling model adapted from cardiac rehabilitation, and delivered by a nurse practitioner with cardiovascular expertise. They postulate this will provide both an appropriate and cost-effective follow-up model.

We hope you enjoy the breadth and depth of this issue's articles.

## Author Contributions

AH prepared the first draft of the editorial. DC reviewed and revised the editorial. Both authors have approved the final version.

## Conflict of Interest

The work of AH is supported by a National Health and Medical Research Council (Australia) Early Career Fellowship (APP1141570) “Premature Cardiovascular Death in Women after hypertensive pregnancy: altering this trajectory.” AH also receives funding in this topic area from the NSW Health Translational Research Grants Scheme. AH is a co-author on 2 of the manuscripts in this Research Topic. AH and DC have professional links to, and collaborations with, a number of authors on manuscripts included in this Research Topic. DC has received support from the British Heart Foundation, Oxford Martin School and the NIHR Oxford Biomedical Research Centre, and the views he expressed are not necessarily those of the funders. These potential conflicts of interest were declared when manuscripts were submitted and neither AH nor DC were involved in reviewing manuscripts where they had a potential conflict of interest.

